# Geometric morphometric analysis of wing size and shape variation in *Aedeomyia catasticta* (Diptera: Culicidae) populations across different regions of Thailand

**DOI:** 10.5455/javar.2026.m1029

**Published:** 2026-03-24

**Authors:** Tanawat Chaiphongpachara, Tawee Saiwichai, Sedthapong Laojun

**Affiliations:** 1 Department of Public Health and Health Promotion, College of Allied Health Sciences, Suan Sunandha Rajabhat University, Bangkok, Thailand; 2 Department of Parasitology and Entomology, Faculty of Public Health, Mahidol University, Bangkok, Thailand

**Keywords:** Mosquito, geometric morphometrics, wing size, wing shape, morphological variation

## Abstract

**Objectives:** *Aedeomyia catasticta* is a mosquito species with relatively limited biological information. Therefore, this study aims to examine the geographical variation of wing shape in *A. catasticta* populations from different regions of Thailand using a landmark-based geometric morphometric approach.

**Materials and Methods:** Samples of *A. catasticta* were collected from each of four regions of Thailand, each with distinct geographical and ecological characteristics: Eastern, Northeastern, Southern, and Western. Wing size was estimated by comparing centroid size (CS) values between populations. Differences in wing shape were assessed using the Mahalanobis distance, and statistical significance was tested using 10,000 permutations.

**Results:** Statistical analyses of wing size indicated significant differences in wing CS among various pairs of populations across different regions, particularly between eastern and western, eastern and southern, and northeastern and western populations (*p* < 0.05). Statistical comparisons of wing shapes also demonstrated that wing shape varied significantly across all four regions (*p* < 0.05).

**Conclusions:** The wing CS and shape of *A. catasticta* populations from different regions of Thailand may have been influenced by regional environmental factors, as indicated by geometric morphometric analyses. These findings improve understanding of regional morphological variation in this understudied mosquito species.

## 1. Introduction

*Aedeomyia* Theobald, 1901, is a genus of small mosquitoes belonging to the family Culicidae, subfamily Culicinae, and tribe Aedeomyiini [1, 2]. It currently comprises seven recognized species, which are classified into two subgenera: *Aedeo myia* (including six species: *A. africana* Neveu-Lemaire, 1906; *A. catasticta* Knab, 1909; *A. madagascarica* Brunhes, Boussès & da Cunha Ramos, 2011; *A. pauliani* Grjebine, 1953; *A. squamipennis* (Lynch Arribálzaga, 1878); and *A. venustipes* (Skuse, 1889)) and Lepiothauma (represented by a single species, *A. furfurea* Enderlein, 1923) [3]. Among these species, four occur in the Afrotropical region (sub-Saharan Africa and Madagascar), one is native to the Australasian region (Australia, New Guinea, and nearby Pacific islands), another is distributed across the Neotropical region (Central and South America, including the Caribbean), and one species is found in both the Oriental region (South and Southeast Asia, extending into southern China and the Malay Archipelago) and the Australasian region [3]. The larvae of *Aedeomyia* species are commonly found in swamps, along the edges of slow-moving streams, and in land ponds with many water plants. These larvae can remain underwater for a long time, sometimes up to 10 min, without surfacing for air. This observation has been associated with their unusually large antennae [1]. Most adult female mosquitoes feed on birds [1]. Previous studies have revealed important avian diseases in *Aedeomyia* mosquitoes, encompassing several arboviruses and protozoa [4, 5]. Even with these studies, current knowledge of the biology and ecology of this genus remains limited.

Thailand is a tropical country with the highest incidence of mosquito-borne diseases worldwide [6]. In addition to this public health concern, the country harbors a relatively high diversity of mosquito species, with approximately 400 species recorded [1, 7]. However, only one species of the genus *Aedeomyia* has been recorded in Thailand, namely *Aedeomyia catasticta* Knab, 1909. Adult *A. catasticta* has unique physical traits, such as thick, wide yellow and white wing scales and large tufts of scales on the mid- and hind femora [1]. Although *A. catasticta* can bite humans, there are no clear reports of it being a vector for human pathogens [1]. *A. catasticta* can breed in a wide range of habitats, such as ponds, swamps, marshes, ditches, pits, floodplains, stream pools, stream banks, seepage areas, and rice fields [1, 2]. In terms of distribution, past studies have reported that *A. catasticta* is found in many regions of Thailand, including the Eastern, Northeastern, Southern, and Western [1, 8]. This mosquito is common in Thailand, but it is challenging to study because it rarely bites people [1, 8].

Geometric morphometrics (GM) has been considered a highly effective technique for examining morphological variation in insects, particularly in wings [9, 10]. Wing GM analysis measures size and shape using two-dimensional coordinates at the intersection points of wing veins [11, 12]. Recently, this technique has been applied to examine regional wing variation in several mosquito vectors in Thailand, including *Armigeres subalbatus* (Coquillett, 1898) [13], *Anopheles baimaii* (Sallum & Peyton, 2005) [14], and *Culex gelidus* (Theobald, 1901) [15]. These results indicate that geographical variation contributes to population-level differences in wing morphology, providing insights into mosquito morphological adaptations and responses to the environment [16].

The environment plays a critical role in shaping both physiological and morphological traits in insects, including mosquitoes [17, 18]. Since environmental conditions are closely linked to geography, factors such as altitude, vegetation type, climatic variables (temperature, rainfall, humidity), and breeding site characteristics can strongly influence wing development [17]. These ecological drivers impose distinct selective pressures across localities, leading to variation in wing size and shape. Understanding the environmental conditions that may affect mosquito wing shape is crucial for describing population dynamics and adaptive responses. Despite its importance, the current study has yet to investigate the impact of geographical and environmental variability on the wing morphology of *A. catasticta* mosquitoes in Thailand.

This study aims to examine the geographical variation in *A. catasticta* wing shape throughout several regions of Thailand using a landmark-based geometric morphometric technique. These findings are expected to provide novel insights into *A. catasticta*, a mosquito species that has not been thoroughly studied in Thailand, by offering comprehensive data on wing morphology across several locations, thereby addressing a critical deficiency in the comprehension of this mosquito species’ environmental adaptations.

## 2. Materials and Methods

### 2.1. Ethical approval

This study was approved by the Suan Sunandha Rajabhat University Institutional Animal Care and Use Committee, Thailand (IACUC No. 66–001/2023).

### 2.2. Study sites and mosquito collection

Specimens of *A. catasticta* were collected from four regions of Thailand: the Eastern, Northeastern, Southern, and Western regions, which represent distinct geographic and ecological zones (Table 1). Trat Province (12°21′07.1″N, 102°24′30.6″E) was selected to represent the Eastern region, Nakhon Ratchasima Province (14°31′35.7″N, 101°21′50.9″E) the Northeastern region, Surat Thani Province (9°13′41.0″N, 99°13′56.8″E) the Southern region, and Kanchanaburi Province (14°07′03.0″N, 99°01′10.4″E) the Western region. Each province exhibits clearly defined geographical and ecological characteristics [19]. Trat is a province in eastern Thailand, bordered by Cambodia and the Gulf of Thailand. The climate is tropical and humid, with rain falling throughout the entire year. The landscape is mostly coastal lowlands with mangrove forests and many small islands [19]. Nakhon Ratchasima is a province in northeastern Thailand on the Khorat Plateau. The terrain is mostly flat, with rolling grasslands, mixed deciduous forests, and scattered farms. The province has a dry season that is rather clear, and it is affected by both the southwest and northeast monsoons [19]. Surat Thani is a province in southern Thailand, bordering the Gulf of Thailand. The weather is tropical and humid, with frequent rain. The terrain is varied, with coastal plains and marshes [19]. Kanchanaburi is a province in Thailand’s western region. There are towering mountains, valleys, and rivers that connect throughout its terrain. It shares a border with Myanmar and has several protected forest areas [6]. Its varied landscape makes it very ecologically rich [6].

**Table 1.** Sampling locations, coordinates, elevation, and number of *Aedeomyia catasticta* specimens used for geometric morphometric analysis.

Region	Sampling location	Latitude / Longitude	Elevation (m)	No. of specimens
Eastern	Khao Saming subdistrict, Khao Saming District, Trat Province	12°21′07.1″N 102°24′30.6″E	36.6 m	33
Northeastern	Moosi subdistrict, Pak Chong district, Nakhon Ratchasima Province	14°31′35.7″N 101°21′50.9″E	387.2 m	12
Southern	Leelet subdistrict, Phunphin District, Surat Thani Province	9°13′41.0″N 99°13′56.8″E	6.6 m	30
Western	Bongti subdistrict, Sai Yok District, Kanchanaburi Province	14°07′03.0″N 99°01′10.4″E	267.3 m	30

**Figure 1.**
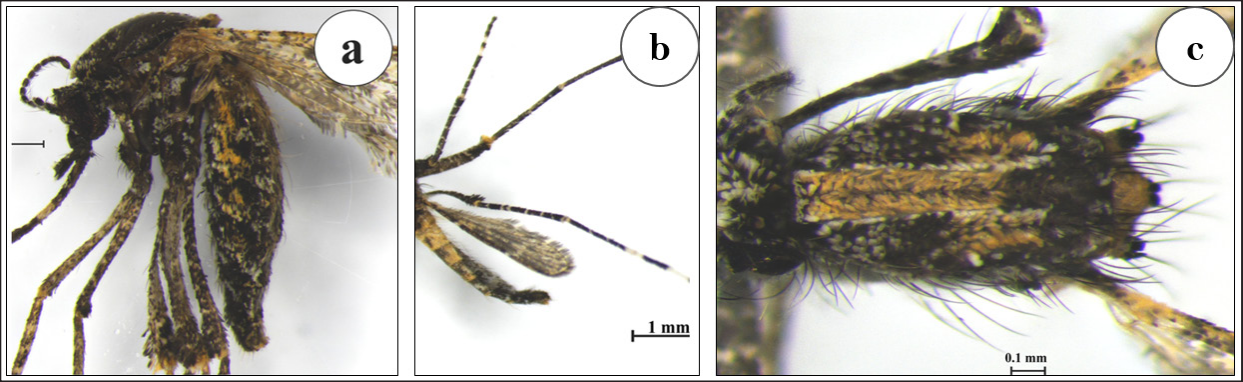
Key morphological diagnostic features of *Aedeomyia catasticta*. Notable characteristics include pale scales on palpal segment 4 and the first antennal flagellomere (a); a distinct dark apical band on the third hind tarsomere, with the fifth tarsomere entirely pale (b); and white scales on the head and scutum forming a prominent pale patch (c).

Collection sites were selected based on previous studies reporting the presence of *A. catasticta* in these areas [8, 19]. Eight CDC light traps with dry ice were used to capture mosquito samples. The traps were hung about 1.5 m above the ground near water sources such as rice paddies, wetlands, and other places with many aquatic plants, which are breeding grounds for *A. catasticta* mosquitoes. From May 1 to June 30, 2024, all samples were collected at night, between 6:00 PM and 6:00 AM, for 7 consecutive nights at each location. This was during the rainy season. We took all the samples in the same season so that seasonal changes wouldn’t affect the features of mosquito wings. Each morning, the trapped samples were removed and euthanized using dry ice at approximately –20°C. All mosquitoes were then morphologically identified using standard taxonomic keys [1]. The distinguishing features of *A. catasticta* are illustrated in Figure 1. Morphologically confirmed *A. catasticta* female specimens were then separated, individually preserved in labeled tubes containing 95% ethanol, and stored at –20°C prior to wing slide preparation. Only females were included because they are the blood-feeding sex and therefore more relevant to epidemiological studies.

### 2.3. Wing preparation and imaging

The identified *A. catasticta* specimens were selected for wing slide preparation. Only specimens with intact right wings were used. Under a Nikon SMZ 800N stereo microscope (Nikon Corp., Tokyo, Japan), the right wings were carefully detached using a fine needle. Each wing was placed at the center of a microscope slide and covered with a coverslip, with Hoyer’s solution applied to secure it in place. The prepared wing slides were then left to dry at room temperature for one week. After drying, the slides were photographed using a digital camera attached to the Nikon SMZ 800N stereo microscope. Each wing image was labeled with its assigned ID code, which could be used to verify additional information about the corresponding specimen.

### 2.4. Landmark digitization and geometric morphometric analysis

**Figure 2.**
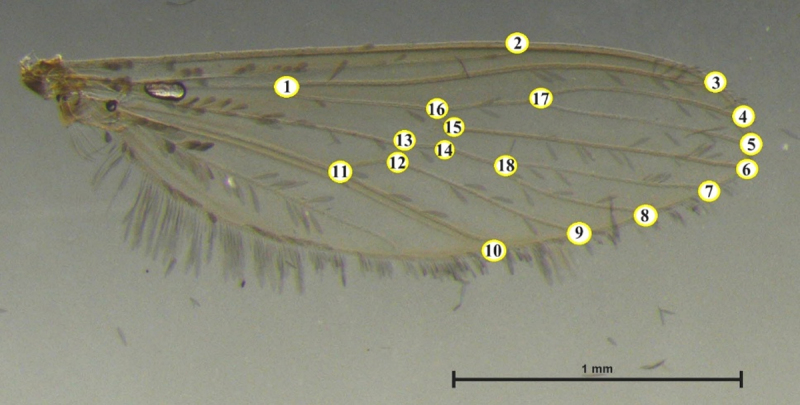
Eighteen landmarks positioned on the wings of *Aedeomyia catasticta*, sequentially arranged according to the numbering from 1 to 18 as shown in the figure, for use in geometric morphometric analysis.

All images of mosquito wings were plotted with 18 landmarks positioned at the intersections of the wing vein lines (Figure 2). The set of landmarks used in this study was selected from previous research on the variability of mosquito wings in Thailand [12, 20]. The 18 landmarks were located at wingline intersection points, where repeatable coordinates could be applied to all samples. We also evaluated the accuracy of landmark digitization using a repeatability test: we randomly selected 30 images, digitized the landmarks twice, and calculated the repeatability index as described by Arnqvist and Mårtensson [21].

Geographical variation in mosquito wings was assessed using size and shape analyses. Wing size was estimated using the centroid size (CS), calculated from the square root of the sum of the squared distances from each landmark to the centroid of the configuration. Statistical differences in CS between *A. catasticta* populations from four regions of Thailand were analyzed using a permutation-based ANOVA with 1,000 replicates and post hoc pairwise comparisons. Landmark digitization and size analysis were carried out in XYOM version 2 [22]. Subsequently, the effect of size on wing shape was examined by performing a multiple regression analysis, with Procrustes coordinates as the dependent variable and CS as the independent variable. The residuals from this regression were used as shape variables in the shape analysis, as they represent shape variation independent of size.

Wing shape variation was assessed using canonical variate analysis (CVA) to identify the most important features distinguishing among groups and to calculate Mahalanobis distances. Statistical differences in Mahalanobis distances between geographic groups were evaluated using a permutation test with 10,000 replicates. The relationships among geographic populations based on wing shape were further examined using a hierarchical clustering tree constructed with the UPGMA algorithm from Mahalanobis distances, with node support assessed by 10,000 bootstrap replicates. Finally, cross-validated classification was performed to assess the accuracy and consistency of assigning individuals to their original regional populations based on wing shape. In all analyses, statistical significance was set at *p* < 0.05. Shape analyses, including CVA and cross-validation, were conducted in MorphoJ version 1.07a [23], and the hierarchical clustering tree was generated in PAST version 4.06b [24].

## 3. Results

A total of 105 *A. catasticta* specimens from Thailand were collected for GM analysis, including 33 from the Eastern region, 12 from the Northeastern region, 30 from the Southern region, and 30 from the Western region. Since the reliability of morphometric data depends on the accuracy of landmark digitization, potential errors must be accounted for. High digitization error can distort shape coordinates, inflate within-group variation, mask true biological differences, and even generate artificial group separation. In this study, the precision of landmark digitization was evaluated, and the repeatability score demonstrated high accuracy, with 98% shape consistency. Consequently, all digitized wing landmark data were used for subsequent size and shape analyses without the need for re-digitizing coordinates.

### 3.1. Size variation

**Figure 3.**
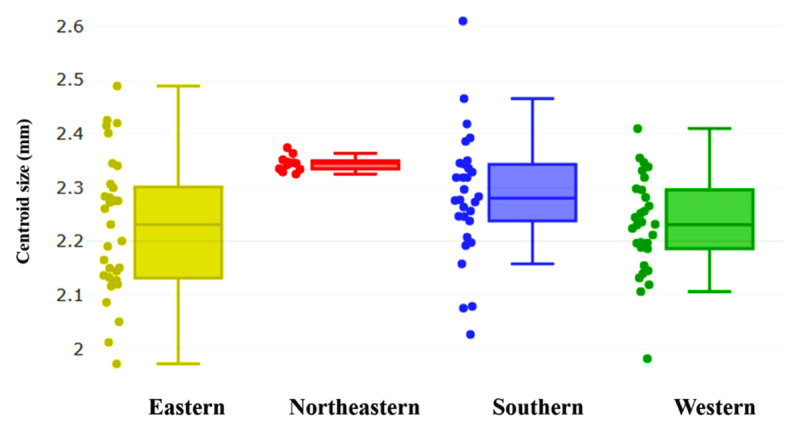
Quantile box plots illustrating centroid size variation of *Aedeomyia catasticta* across four regions of Thailand: Eastern (yellow, *n* = 33), Northeastern (red, *n* = 12), Southern (blue, *n* = 30), and Western (green, *n* = 30). The horizontal line within each box represents the median, while the box edges indicate the 25th and 75th percentiles.

The variation in wing CS of *A. catasticta* across four different regions of Thailand is illustrated in Figure 3. The Northeastern population has the smallest dispersion and a narrow interquartile range of wing centroid size, indicating high homogeneity among samples. In contrast, the Southern, Eastern, and Western populations have wider dispersion and broader centroid size ranges, indicating higher intraspecific variation. The mean wing CS of *A. catasticta* varied among the four regional populations in Thailand, as shown in Table 2. The highest mean CS was observed in the Northeastern population (2.345 mm), followed by the Southern (2.284 mm), Western (2.229 mm), and Eastern (2.227 mm) populations, respectively. The CS range was narrowest in the Northeastern population (2.325–2.374 mm), indicating low variability, whereas the Southern population showed the widest range (2.027–2.610 mm). Statistical comparisons of mean wing CS revealed significant differences among some regional populations (*p* < 0.05), including the Eastern and Western populations, the Eastern and Southern populations, and the Northeastern and Western populations (Table 2).

**Table 2.** The mean wing centroid size and statistical differences of *Aedeomyia catasticta* among four regions of Thailand: Eastern, Northeastern, Southern, and Western.

Region	Mean CS (mm)	Range (Min–Max)	Variance	SD
Eastern	2.227^a^	1.972–2.489	0.016	0.127
Northeastern	2.345^b^	2.325–2.374	0.001	0.014
Southern	2.284^b,c^	2.027–2.610	0.014	0.117
Western	2.229^a^	1.982–2.410	0.008	0.089

Note: Superscript letters (a, b, c) indicate groups that are significantly different from one another.

The influence of wing size on shape variation was assessed using a multivariate regression of shape variables for CS. The results indicated that 5.96% of the total wing shape variation was attributable to size and was statistically significant (*p* < 0.05), as determined by a permutation test with 10,000 randomizations. This confirms a significant relationship between size and shape. Consequently, the residuals from this regression were used as the final shape variables in the shape analysis to avoid confounding by size.

### 3.2. Shape variation

**Figure 4.**
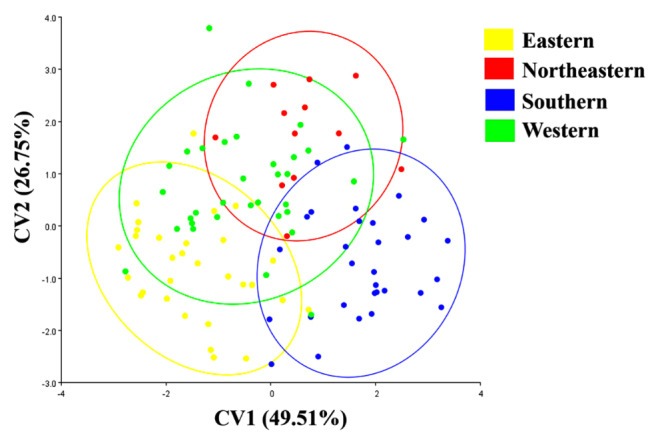
Scatter plot of the first two canonical variate axes (CV1 and CV2) from canonical variate analysis (CVA), illustrating wing shape variation in *Aedeomyia catasticta* from four regions of Thailand: Eastern (yellow), Northeastern (red), Southern (blue), and Western (green). CV1 and CV2 account for 49.51% and 26.75% of the total variation, respectively. Each ellipse represents the 90% confidence region for its corresponding population.

Wing shape differentiation among regional populations of *A. catasticta* was examined using CVA based on allometry-free shape variables (Figure 4). The analysis yielded three canonical variates, with the first two accounting for the majority of the total wing shape variation: 49.51% for the first canonical variable (CV1) and 26.75% for the second (CV2), together explaining 76.26% of the overall variation. The scatterplot indicated some overlap among the four regions. Mahalanobis distance comparisons (Table 3) revealed statistically significant differences in wing shape among all four regional populations of *A. catasticta* (*p* < 0.05). The largest pairwise distance was between the Eastern and Northeastern populations (3.496), followed by the Eastern and Southern (3.250) and the Northeastern and Southern populations (3.246), while the smallest distance was between the Eastern and Western populations (2.414).

**Table 3.** Mahalanobis distances (below diagonal) and *p*-values (above diagonal) for wing shape differentiation in *Aedeomyia catasticta* among four regions of Thailand: Eastern, Northeastern, Southern, and Western.

Distances/*p*-values	Eastern	Northeastern	Southern	Western
Eastern	–	**< .0001**	**< .0001**	**< .0001**
Northeastern	3.496	–	**< .0001**	**< .0001**
Southern	3.250	3.246	–	**< .0001**
Western	2.414	3.119	2.790	–

Bold *p*-values (< 0.05) indicate statistically significant differences.

**Figure 5.**
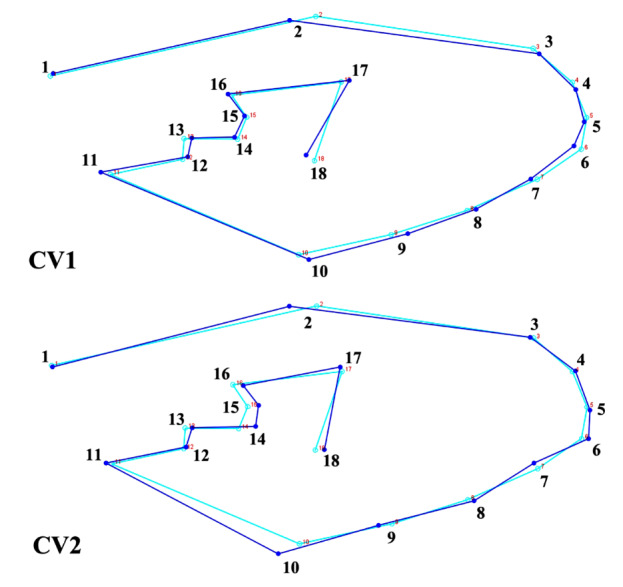
Wireframe diagrams illustrating deviations in landmark positions from the overall mean wing shape of *Aedeomyia catasticta* from four regions of Thailand along the first and second canonical variate (CV) axes (CV1 [top] and CV2 [bottom]). The light blue outline represents the mean shape configuration, while the dark blue outline depicts the directional shifts in landmark positions corresponding to shape variation along each axis. Please remove the little red numbers in this figure. They are distracting and not necessary.

The wing wireframe diagrams in Figure 5 illustrate shape changes along the main axes of wing shape variation among populations from four different regions of Thailand. The CV1 accounted for the greatest portion of shape differentiation, showing a pronounced shift in the anterior part of the wing, particularly at landmarks 2 to 7 and landmark 10 (Figure 5).

Additional shape changes were observed at the wing base, especially at landmarks 12, 13, 17, and 18. The CV2 captured additional variation, mainly concentrated in the posterior-mid region of the wing, with noticeable shifts at landmarks 10–18 (Figure 5).

### 3.3. Cluster analysis of wing shape variation

**Figure 6.**
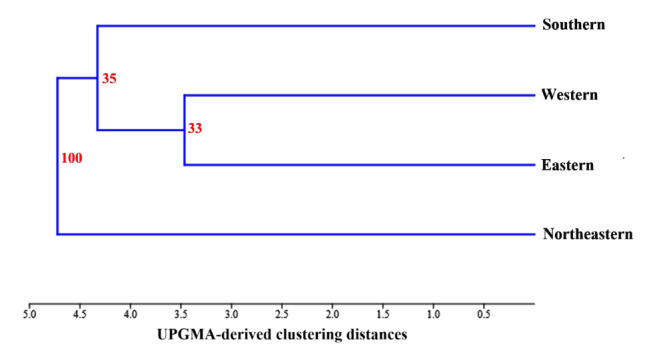
Hierarchical clustering tree based on wing shape variation in *Aedeomyia catasticta* from four regions of Thailand: Eastern, Northeastern, Southern, and Western. The tree was constructed using the UPGMA algorithm derived from Mahalanobis distances, and the numbers on the nodes indicate bootstrap support percentages based on 10,000 replicates.

A hierarchical clustering tree, constructed using Mahalanobis distances and supported by 10,000 bootstrap replicates, revealed distinct patterns of wing shape grouping among *A. catasticta* populations from different regions of Thailand (Figure 6). Populations from the Eastern and Western regions exhibited the greatest similarity in wing shape, clustering closely together with bootstrap support of 33% and being further grouped with the Southern population, with bootstrap support of 35%. In contrast, the Northeastern population showed the most distinct wing shape, forming a separate cluster with strong bootstrap support at 100%.

### 3.4. Classification accuracy of regional wing shape differentiation

Cross-validated classification was used to assess the consistency and distinctiveness of wing shape variation among *A. catasticta* populations from four regions of Thailand. The overall classification success rate was 55.24%. All four regional populations showed relatively similar correct classification rates. These ranged from 53.33% in the Southern population to 58.33% in the Northeastern population (Table 4).

**Table 4.** Cross-validated classification results showing classification accuracy (%) of *Aedeomyia catasticta* from four regional populations in Thailand.

Region	% Correctly classified	No. correctly classified / total specimens
Eastern	54.55	18 / 33
Northeastern	58.33	7 / 12
Southern	53.33	16 / 30
Western	56.67	17 / 30
Total	55.24	58 / 105

## 4. Discussion

This study is the first to assess regional variation in wing morphology of *A. catasticta* in Thailand. Our analysis revealed significant differences in wing morphology between populations from different regions. Such differences reflect the influence of environmental or geographical factors on mosquito physiology, consistent with previous studies that found geographical differences affect other mosquito vectors such as *Aedes albopictus* (Skuse, 1894) and *Aedes scapularis* (Rondani, 1848) [25]. An analysis of wing size in A. catasticta mosquitoes from four regions of Thailand revealed statistically significant differences among several population pairs (Table 2). We hypothesize that these variations in wing size result from differences in environmental conditions across regions, such as temperature, humidity, and rainfall [14]. Previous research has demonstrated that mosquito wing size can be considerably influenced by the quality of the larval breeding site, such as its density [26]. For instance, a study in Iquitos, Peru, examined the relationship between breeding site and wing size in *Aedes aegypti*. The results indicated that the average wing length was positively correlated with the presence of larvae, the container-filling method, container diameter, and larval density [26]. Similar to *Anopheles gambiae* (s.s.), the larval environment, particularly food availability and temperature, significantly influenced body size and lifespan [27]. The wing length decreased as temperature increased and food availability decreased, suggesting that larval conditions have a direct impact on adult body size [27]. In the present study, the Northeastern population exhibited the highest mean wing CS. It is possible that the sampling site’s proximity to Khao Yai National Park, which supports various freshwater ecosystems, such as ponds, wetlands, marshes, ditches, pits, stream beds, stream banks, and seepage areas, serves as a suitable breeding ground for *A. catasticta*. This is consistent with previous studies comparing the wing size of *Aedes albopictus* between island and mainland populations in Ranong Province, Thailand, which reported that mosquitoes from mainland areas had larger wings. This is due to the diversity and abundance of habitats, which support the large number of larvae found in those areas [28].

Previous studies on other mosquito species have also indicated that temperature affects wing size, with populations in warmer climates tending to be smaller than those in cooler climates [29]. An examination of the average annual temperatures in 2023 in our four study regions revealed only slight variation in temperature, with the average temperature in Trat Province (Eastern region) at approximately 27.4°C, followed by Nakhon Ratchasima Province (Northeastern region) at 27.7°C, Kanchanaburi Province (Western region) at 27.5°C, and Surat Thani Province (Southern region) at 27.1°C [30]. Therefore, our study results cannot definitively conclude that temperature has a significant influence on wing size. However, we hypothesize that the difference in wing size may not be due to temperature but rather to other environmental variables, such as habitat quality, resource availability, and species-specific ecological adaptation, playing a more significant role.

An examination of wing shape variation in *A. catasticta* across four regions revealed statistically significant differences between all populations (Table 3), clearly indicating that localized environmental conditions influence wing morphology. This result is consistent with previous studies on several major mosquito vectors in Thailand, including *A. subalbatus* [13], *A. baimaii* [14], and *C. gelidus* [15], which reported regional differences in wing shape that may also be driven by environmental factors. Such morphological variation likely reflects biological adaptations to localized selective pressures. Analysis of wing wireframe diagrams shows that shape variation is distributed across nearly the entire wing, with particularly pronounced changes along the lower wing margin and the internal outline. These areas are critical to flight dynamics, implying that flight performance in *A. catasticta* may vary among regional populations [13]. Nonetheless, this hypothesis warrants further investigation in future studies.

In addition, the results of the hierarchical cluster analysis were consistent with those of the CVA and Mahalanobis distance pairwise comparisons, all of which revealed significant differences in wing shape among regional populations. The northeastern population appears to exhibit the greatest shape specificity, as indicated by cross-validated classification results, which show it has the highest correct classification accuracy (58.33%). The northeastern region (e.g., Nakhon Ratchasima Province) is located on the Khorat Plateau, a unique topographic region with physical barriers, such as mountain ranges, that may limit gene flow and promote population specificity [15, 31, 32, 33]. However, the analysis results for the northeastern population may require careful interpretation, as this region has only 12 samples, which may lead to disproportionate analysis. Therefore, while preliminary results suggest that *A. catasticta* populations in the northeastern region may exhibit distinctly different wing characteristics from other regions, these findings should be verified with larger sample sizes and supplemented with genetic analysis.

The sample size from the Northeastern region was small and may not permit definitive conclusions. Nevertheless, significant differences were also detected among the other three regional populations, specifically the Eastern, Southern, and Western regions, providing preliminary evidence of geographic variation within the species. The physiological differences in wings between these regions may be influenced by the diverse ecological environments across Thailand, including variations in climate, hydrology, vegetation, and host availability, all of which can affect morphological changes in mosquito populations [15, 32, 33].

## 5. Conclusions

This study provides the first report of regional variation in wing morphology of *Aedeomyia catasticta* in Thailand. GM analysis revealed clear morphological differentiation among regional populations, suggesting that environmental or geographic factors may influence wing traits. Variations in both wing size and shape appear to be shaped by local environmental conditions. These study results provide important information about the morphological changes in the wings of *A. catasticta*, a previously understudied mosquito species. Although *A. catasticta* has not yet been reported as a disease vector, its blood-feeding behavior (primarily on animals and sometimes on humans) suggests a potential role as a vector for zoonotic pathogens.

## Data Availability

The data presented in this study are available from the corresponding author upon reasonable request.

## List of abbreviations

ANOVA, analysis of variance; CS, centroid size; CV, canonical variable; CVA, canonical variate analysis; GM, geometric morphometrics.

## References

[1] Rattanarithikul R, Harrison BA, Panthusiri P, Peyton EL, Coleman RE (2006). Illustrated keys to the mosquitoes of Thailand III. Genera *Aedeomyia, Ficalbia, Mimomyia, Hodgesia, Coquillettidia, Mansonia* and *Uranotaenia*. Southeast Asian J Trop Med Public Health.

[2] Boehmler M (2022). *Aedeomyia* (*Aedeomyia*) *squamipennis*—new genus and species county record for Monroe county, Florida, USA. J Am Mosq Control Assoc.

[3] Harbach RE (2025). Mosquito Taxonomic Inventory. Mosq Taxon Invent Valid Species List.

[4] Loaiza JR, Miller MJ (2013). Seasonal pattern of avian *Plasmodium*-infected mosquitoes and implications for parasite transmission in central Panama. Parasitol Res.

[5] Tantely LM, Cêtre-Sossah C, Rakotondranaivo T, Cardinale E, Boyer S (2017). Population dynamics of mosquito species in a West Nile virus endemic area in Madagascar. Parasite.

[6] Kwansomboon N, Chaumeau V, Kittiphanakun P, Cerqueira D, Corbel V, Chareonviriyaphap T (2017). Vector bionomics and malaria transmission along the Thailand-Myanmar border: a baseline entomological survey. J Vector Ecol.

[7] Tisgratog R, Tananchai C, Juntarajumnong W, Tuntakom S, Bangs MJ, Corbel V (2012). Host feeding patterns and preference of *Anopheles minimus* (Diptera: Culicidae) in a malaria endemic area of western Thailand: Baseline site description. Parasit Vector.

[8] Chaiphongpachara T, Changbunjong T, Laojun S, Nutepsu T, Suwandittakul N, Kuntawong K (2022). Mitochondrial DNA barcoding of mosquito species (Diptera: Culicidae) in Thailand. PLoS One.

[9] Laojun S, Changbunjong T, Bunchu N, Chaiphongpachara T (2026). Microbial communities and wing variation associated with ectoparasitic mites in medically important *Mansonia* mosquitoes (Diptera: Culicidae) from coconut plantation habitats in central Thailand. Acta Trop.

[10] Aupalee K, Srisuka W, Limsopatham K, Sanit S, Takaoka H, Saeung A (2024). Reliability of wing morphometrics for species identification of human-biting black flies (Diptera: Simuliidae) in Thailand. Parasit Vectors.

[11] Jaramillo-O N, Dujardin JP, Calle-Londoño D, Fonseca-González I (2015). Geometric morphometrics for the taxonomy of 11 species of *Anopheles* (*Nyssorhynchus*) mosquitoes. Med Vet Entomol.

[12] Laojun S, Changbunjong T, Chaiphongpachara T (2023). Evaluation of modern techniques for species identification of *Lutzia* mosquitoes (Diptera: Culicidae) in Thailand: Geometric morphometrics and DNA barcoding. Insects.

[13] Laojun S, Changbunjong T, Chaiphongpachara T (2024). Population genetic structure and wing geometric morphometrics of the filarial vector *Armigeres subalbatus* (Diptera: Culicidae) in Thailand. Acta Trop.

[14] Laojun S, Changbunjong T, Chaiphongpachara T (2024). Insights into the mitochondrial cytochrome oxidase I (*mt-COI*) gene and wing morphometrics of *Anopheles baimaii* (Diptera: Culicidae) in malaria-endemic islands of Thailand. Parasitol Res.

[15] Chaiphongpachara T, Laojun S, Changbunjong T, Wichit S, Villarroel PMS (2024). Demographic inference from the mt-DNA *COI* gene and wing geometry of *Culex gelidus* (Diptera: Culicidae), an important vector of Japanese encephalitis in Thailand. Acta Trop.

[16] Louise C, Vidal PO, Suesdek L (2015). Microevolution of *Aedes aegypti*. PLoS One.

[17] Vargas RM, Tsunoda T, Noda J, Bousses P, Nguyen TY, Hasebe F (2021). Shape relatedness between geographic populations of *Culex tritaeniorhynchus* the primary vector of Japanese encephalitis virus: A landmark study. Infect Genet Evol.

[18] Vargas REM, Phumala-Morales N, Tsunoda T, Apiwathnasorn C, Dujardin JP (2013). The phenetic structure of *Aedes albopictus*. Infect Genet Evol.

[19] Royal Forest Department (2023).

[20] Laojun S, Changbunjong T, Kaewthamasorn M, Charnwichai P, Kaewmee S, Wichit S (2025). Accurate identification of medically important *Aedes* mosquitoes (Diptera: Culicidae) in Thailand through DNA barcoding, wing geometric morphometrics, and machine learning. Curr Res Parasitol Vector Borne Dis.

[21] Arnqvist G, Mårtensson T (1998). Measurement error in geometric morphometrics: Empirical strategies to assess and reduce its impact on measures of shape. Acta Zool Acad Sci Hung.

[22] Dujardin S, Dujardin JP (2019). Geometric morphometrics in the cloud. Infect Genet Evol.

[23] Klingenberg CP (2011). MorphoJ: An integrated software package for geometric morphometrics. Mol Ecol Resour.

[24] Hammer Ø, Harper DAT, Ryan PD (2001). PAST: Paleontological statistics software package for education and data analysis. Palaeontol Electron.

[25] Oliveira-Christe R, Wilke ABB, Marrelli MT (2020). Microgeographic wing-shape variation in *Aedes albopictus* and *Aedes scapularis* (Diptera: Culicidae) populations. Insects.

[26] Schneider JR, Morrison AC, Astete H, Scott TW, Wilson ML (2004). Adult size and distribution of *Aedes aegypti* (Diptera: Culicidae) associated with larval habitats in Iquitos, Peru. J Med Entomol.

[27] Barreaux AMG, Stone CM, Barreaux P, Koella JC (2018). The relationship between size and longevity of the malaria vector *Anopheles gambiae* (s.s.) depends on the larval environment. Parasit Vectors.

[28] Laojun S, Sontigun N, Chaiphongpachara T (2024). Influence of insular conditions on wing phenotypic variation in two dominant mosquito vectors *Aedes albopictus* and *Armigeres subalbatus* (Diptera: Culicidae), in the border archipelagos of Thailand. Med Vet Entomol.

[29] Laojun S, Chaiphongpachara T (2025). Phenotypic and genetic variation of *Aedes albopictus* (Diptera: Culicidae) in Thailand and its global relationships: insights from wing morphometric and mitochondrial *COI* gene analyses. Med Vet Entomol.

[30] Weather Spark (2025). The typical weather anywhere on Earth.

[31] Chaiphongpachara T, Laojun S (2019). Annual variability of wing morphology in *Culex sitiens* Wiedemann (Diptera, Culicidae) mosquito vectors from the coastal area of Samut Songkhram Province, Thailand. J Parasitol Res.

[32] Suesdek L (2019). Microevolution of medically important mosquitoes—a review. Acta Trop.

[33] Laojun S, Changbunjong T, Chaiphongpachara T (2025). Intraspecific genetic variation in the lymphatic filariasis vector *Mansonia dives* (Diptera: Culicidae) in Thailand: Hidden species or genetically divergent populations?. Acta Trop.

